# Translating research to policy at the NCSE 2017 symposium “Microbiology of the Built Environment: Implications for Health and Design”

**DOI:** 10.1186/s40168-018-0552-y

**Published:** 2018-09-15

**Authors:** Ashleigh Bope, Mark H. Weir, Amy Pruden, Michael Morowitz, Jade Mitchell, Karen C. Dannemiller

**Affiliations:** 1 Environmental Science Graduate Program, Ohio State University, Columbus, OH 43210 USA; 2 Department of Civil, Environmental & Geodetic Engineering, College of Engineering, Ohio State University, Columbus, OH 43210 USA; 3 Division of Environmental Health Sciences, College of Public Health, Ohio State University, Columbus, OH 43210 USA; 4 Department of Civil & Environmental Engineering, Virginia Tech, Blacksburg, VA 24060 USA; 5 Department of Surgery, University of Pittsburgh School of Medicine, Pittsburgh, PA 15224 USA; 6 Department of Biosystems and Agricultural Engineering, Michigan State University, East Lansing, MI 48823 USA

**Keywords:** Microbiome, Fungi, Bacteria, Health, Exposure, Hospital, Design, Plumbing, Antibiotic resistance, Risk

## Abstract

Here, we summarize a symposium entitled “Microbiology of the Built Environment: Implications for Health and Design” that was presented at the National Council for Science and the Environment (NCSE) 17th National Conference and Global Forum in January 2017. We covered topics including indoor microbial exposures and childhood asthma, the influence of hospital design on neonatal development, the role of the microbiome in our premise (i.e., building) plumbing systems, antibiotic resistance, and quantitative microbial risk assessment. This symposium engaged the broader scientific and policy communities in a discussion to increase awareness of this critical research area and translate findings to practice.

## Background

We spend 90% of our time indoors [1] where we are exposed to a complex range of microbial communities. Microbial communities influence our health in ways that are only beginning to be understood [2]. The Microbiology of the Built Environment (MoBE) program recently provided a platform for catalyzing a new field of inquiry at the interface of human and built environment microbiomes. Through better understanding the indoor microbiome and the factors that control it, we can begin to link building design choices with exposures, human health, and disease.

We convened a symposium at the recent National Council for Science and the Environment (NCSE) 17th National Conference and Global Forum (Washington D.C., 24–26 January 2017), which focused this year on the theme of “Science, Policy, and the Environment: Integrating Environment and Health.” Our symposium was entitled “Microbiology of the Built Environment: Implications for Health and Design” and brought together scientists, engineers, and practitioners in the field to promote discussion across disciplines towards addressing implications of microbiology on building design and infrastructure, human health, and risk assessment. Representative MoBE topics included linking microbial exposure to human health; understanding the impact of the built environment on early gut bacterial colonization of newborn infants; improving water infrastructure and plumbing management in a manner that correspondingly improves public health; developing risk assessment models for predicting emergence of antibiotic resistance associated with antibiotic use in livestock; and applying quantitative microbial risk assessment (QMRA) modeling to design resilient water systems.

Ten years prior, in 2007, the 7th NCSE Conference explored the connection between human health and the environment. Over this past decade, revolutionary advances in DNA sequencing, environmental microbiology, and microbiome sciences have been made, fueling pioneering research and new insights. This presented the opportunity at the 17th NCSE Conference to explore and reflect upon the complex and interconnected challenges where health and the environment meet and highlight emerging research and policy. The presentations featured at the symposium highlighted how recent advances may be channeled towards a better understanding of the implications for public health and building/infrastructure design. The following subsections of this article are outlined based on the topic areas covered by the symposium, with key elements of the discussion that followed highlighted.

## Microbiology of the Built Environment: implications for childhood asthma

Asthma is a common chronic disease that affects 8% of the total U.S. population and costs an estimated $56 billion per year [3, 4]. Persons of all ages are affected by asthma, and the disease may start in childhood. Asthma control in children can be influenced by various environmental factors. For instance, exposure of asthmatics to dampness and mold in housing costs $3.5 billion per year in the USA [5]. Much of indoor exposure to microorganisms originates from floor dust, which is resuspended as occupants move in the indoor environment [6].

Composition of the indoor microbiome was associated with asthma severity in a study from an urban/suburban cohort of asthmatic children in Connecticut and Massachusetts, USA [7]. Indoor fungal and bacterial exposures were measured from house dust and asthma control was monitored over the following month. Associations between exposure and asthma severity differed by atopic (allergic) status. Asthma severity in atopic children was associated with fungal community composition, while asthma severity in nonatopic children was associated with total fungal concentration. Overall, these results demonstrated that different aspects of microbial communities may be important to consider in different populations.

Exposure to microbes associated with a damp environment may be especially harmful to asthmatics. A laboratory chamber study was used to demonstrate that elevated moisture conditions > 80% equilibrium relative humidity may facilitate microbial growth within house dust itself [8]. This additional growth increases microbial concentrations in dust, and this has the potential to substantially contribute to human exposure, especially when dust is resuspended. Thus, exposures to indoor microbes need to be considered in building design and further highlights the need for moisture control in buildings.

## Microbiology of the Built Environment: implications for hospital design

Hospital environments present a unique built environment type in which there is continuous turnover of input of microbes associated with patients and their visitors coupled with inhabitants that have a relatively higher susceptibility to microbial illness. Of significant concern is that microbes have been revealed to exist in sites engineered to be sterile or near sterile, such as high-risk hospital wards [9, 10]. Studies show that a room’s function or architecture dictates the microbiome in the built environment [9, 11]. The exact mechanism of exchange between the human microbiome and the built environment microbiome remains unclear, but observing the enrichment for human pathogens is an obvious concern in a hospital setting, especially in the Neonatal Intensive Care Unit (NICU) containing low birthweight babies. Infants are well suited for building an understanding of microbiome exchange between humans and buildings because in utero infants are considered sterile or near-sterile [9, 12]. These vulnerable infants are especially susceptible to environmental influences.

It has been estimated that at least 38% of all Intensive Care Unit (ICU) outbreaks could be attributed to microbial sources within the ICU environment. Sources of infective agents include equipment, personnel, infant incubators, sink drains, soap dispensers, thermometers, and baby toys and even agents in the air [9, 13–19]. Using next generation DNA sequencing technologies, analysis of reconstructed genomes can help inform our understanding of how organisms are able to persist in the NICU. In a time-series study of infants, it was observed that antibiotic resistance genes were found in fecal samples [9]. These adaptations make organisms better equipped to withstand constant sterilization and cleaning and the broad use of antibiotics. The current understanding of the interaction between infants and their environment points to a scenario in which microbes are introduced from a source in the built environment, they then thrive in the gut and finally are redistributed into the immediate environment. This creates a cycle of microbes being continuously colonized in both the environment and the infant [9, 12]. These observations should be considered when deciding on changes in practice that can better adapt to this constant cycle as infants come and go from the NICU that ultimately leads to better health outcomes for vulnerable infants.

## Microbiology of the Built Environment: implications for premise plumbing

Premise (i.e., building) plumbing consists of the pipes within households and buildings, including the portion of the service line delivering municipal water beyond the property line up to the various points of use within the building, such as faucets, shower heads, dishwashers, laundry machines, and ice makers. While tap water is often thought to be essentially a “germ free” environment, in reality, premise plumbing provides a rich microbial ecological niche. A major driver of interest in premise plumbing microbiomes is *Legionella pneumophila*, the primary causative agent of Legionnaires’ disease, which is now the number one source of tap-water-related disease outbreak in the USA and other developed countries [20]. In addition to *L. pneumophila*, other opportunistic pathogens growing in premise plumbing, such as nontuberculous mycobacteria (NTM), are also of growing concern. Such organisms share several features that make them adaptive to the tap water environment, including their relative tolerance of chlorine and other disinfectants, hydrophobicity, residence in biofilms, and ability to survive and even multiply when preyed upon by amoebae [21].

Since Legionnaires’ disease originally gained attention following the first identified outbreak in 1976 and NTM was recognized as an opportunistic infection associated with the AIDS epidemic in the 1980s, microbiome science is now beginning to shed new light on the factors that trigger the growth of these organisms in premise plumbing [22, 23]. The range of tolerance to varying temperatures and disinfectant levels of opportunistic pathogens have been fairly well-studied under controlled conditions in the laboratory, and now, recent research has made it possible to extend observations to real conditions encountered in the field. For example, it is important to recognize that the water heater set point is not equivalent to the actual temperatures experienced most of the time at the tap. Rhoads et al. [24] used a pilot-scale pipe rig to demonstrate that while elevated hot water tank temperatures (i.e., > 58 °C) were generally beneficial for the limiting colonization and growth of *Legionella*, if the temperature is not quite hot enough (i.e., 51 °C), then the heat shock and recovery experienced at the distal taps can select for and enrich *Legionella*, presumably by killing off competing bacteria. Further, whether the pipes are plumbed with up or down flow influences convective mixing and nutrient delivery and had an effect on the kinds of microbes colonizing the taps and the suitability for *Legionella* growth [25]. Pipe materials, such as commonly used copper and plastic materials, can also have an effect on the ability of *Legionella* to colonize [26, 27]. Such research opens the door to very practical engineering design controls, such as temperature settings, flow rates, and pipe materials, for limiting the proliferation of pathogens and protecting public health.

Looking into the future, potentially of even greater importance than opportunistic pathogens, will be building an understanding of how water system design influences the composition of the tap water microbiome. Ji et al. [28] noted that identical plumbing rigs, with flow cycles simulating daily use and stagnation cycles and triplicate pipes for comparison, harbored distinct microbiomes depending on the municipal water supply fed to the systems. Further, using the same hot water plumbing rigs described above used for the study of *Legionella* [14, 15], it was found that the water heater tank set point and plumbing configurations also distinctly shapes the kinds of microbes occurring at the tap [29]. As our understanding of the human microbiome advances, research along these lines can aid in identifying how water systems can contribute to a “healthy” or “unhealthy” indoor microbiome and identify means of intentional engineering control.

## Microbiology of the Built Environment: implications for quantitative risk analysis

QMRA was developed initially as a modeling paradigm in the 1980s. The ability to estimate health risks based on exposure to a set of hazards has always been of interest. For instance, Ancient Rome developed an effort to catalog all poisonous plants in the empire [30]. Infectious disease investigations and research gained new interest with the understanding of the germ theory of disease and the explosion in epidemiology since John Snow first linked cholera outbreaks to contaminated well water. What was consistently missing in infectious disease studies and research was a means of predictive power that accounts for the mechanisms of disease in humans. With the development of the mechanistic microbial dose response models in 1983 [31], QMRA evolved as a predictive infectious disease research and analysis tool.

With the bioterror attacks in 2001, QMRA was found to be an invaluable decision analytics tool and the subsequent foundation of the Center for Advancing Microbial Risk Assessment (CAMRA) provided a central location for QMRA research and practice. CAMRA introduced the beginnings of QMRA as a trans-disciplinary science, moving it beyond the useful modeling paradigm it had always been.

**Fig. 1:**
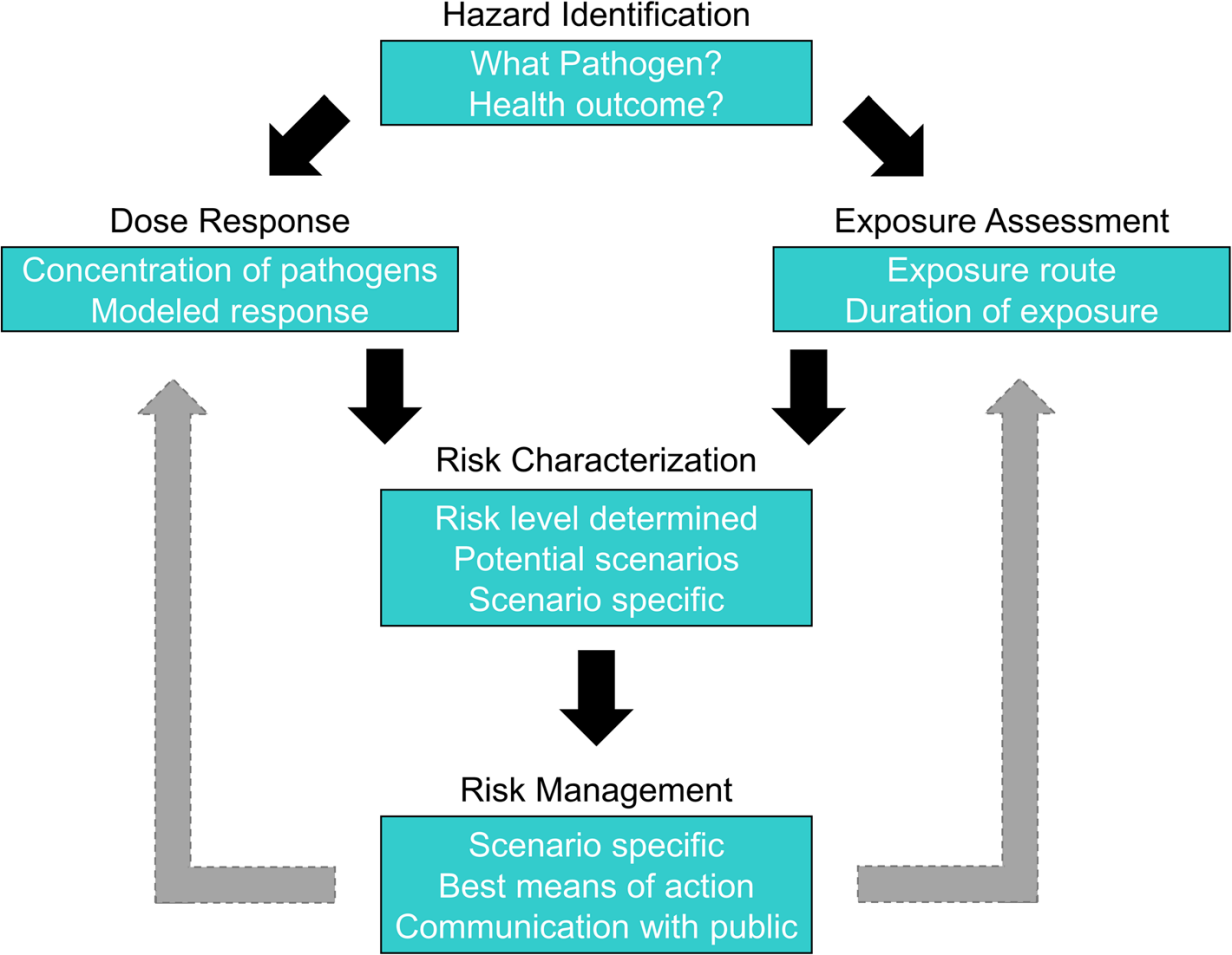
Advanced QMRA paradigm

Figure 1 shows an advanced QMRA paradigm that was developed within CAMRA and has continued use in research and practice. Bi-directional risk communication in the new paradigm shown in Fig. 1 is important, as we are engaged in a computational science that is predictively modeling or estimating health risks from infectious disease agents.

QMRA models can influence engineering design recommendations. A specific case study of a cryptosporidiosis outbreak in a recreational spray park demonstrates the use of QMRA to inform design and remediation [32]. Indoor spaces can also be modeled with QMRA to determine complex exposures and indoor space contamination risks [33].

The concept of using MoBE data in a QMRA model has some limitations and knowledge gaps. A fundamental challenge to incorporating MoBE into QMRA are the dynamics of microbial communities in general. While there are numerous examples, one key is the viable but non-culturable (VBNC) state that some microbes tend to adopt in response to stressful conditions. VBNC is a term ascribed to microorganisms (most commonly bacteria and protozoa) that cannot replicate on culture media, but are not completely killed and thus they can still enter the human host (or other animal host) and cause harm. VBNC microbes cannot, by definition, be detected in culture. While they are detected in molecular datasets, their DNA cannot be distinguished from that coming from dead cells either, thus creating a methodological conundrum.

Use of MoBE data in QMRA requires investment in research primarily on two fronts. First, the ability to predict phenotypic characteristics from genotypic data. This capability will allow for the strength of the data in MoBE to be leveraged to predict infectivity and phenotypic structures required for human infection, a potential answer to the VBNC question as well. Second is the paradigm of QMRA being adapted to accept this new type of data that may not contain viability information. This will allow the fields to evolve together to improve health risk estimation using the most up-to-date data from state of the science technologies.

## Microbiology of the Built Environment: implications for antibiotic resistance

Antibiotic resistance is potentially the greatest public health challenge of the twenty-first century. Antibiotic resistance occurs when the pathogenic bacteria develop virulence factors that prevent them from being susceptible to antibiotics intended to kill them. In general, antibiotic resistant pathogens tend to be observed in the clinical setting within 10 to 20 years of discovery and production of a new antibiotic [34]. Bacteria become resistant because they are extremely versatile, evolve on a much faster timescale than humans, and have the ability to share antibiotic resistance genes (ARGs) with each other via horizontal gene transfer. Antibiotic resistance is increasingly being viewed as an environmental concern given that resistant pathogens may escape sewage treatment or livestock facilities and wind up in drinking water. Additional control measures may be prudent, particularly water treatment and other barriers to the spread of ARGs to indoor exposures via tap water, aerosols, or food. In plumbing environments, there is interest in how the water chemistry, especially from alternative sources such as recycled water, may influence the propensity for biofilms to harbor antibiotic resistant bacteria and ARGs [35, 36].

As human exposures to microorganisms in the environment are beginning to be better understood, it is important to consider the effects of antibiotic resistance on human health. According to the Center for Disease Control and Prevention each year in the USA at least 2 million people acquire serious infections involving bacteria that are resistant to one or more antibiotics designed to treat the infections. Of these, more than 23,000 people die each year as a direct result of antibiotic-resistant infection [37], and even more die from conditions that are complicated by a resistant infection [38]. Antibiotic resistance is an environmental, economic, and public health concern that spans international borders. QMRA modeling may be able to help guide decision making associated with antibiotic resistance concerns. There are major limitations to applying this approach to antibiotic resistance including data gaps related to horizontal gene transfer in the environment and in the guts of humans and animals. Additionally, dose-response models are not available to mathematically describe the relationships between the risk of infection, illness or death and given exposure doses or numbers of pathogens [39].

The use of antibiotics is a critical factor leading to the development and spread of antimicrobial resistance. Of significant concern is that up to 50% of prescribed antibiotics may not be needed or are not effective in the dosage prescribed [37]. In addition, antibiotics are widely used in agriculture, and while recent measures have been taken to prohibit their use solely for growth promotion in the USA [40], it will be important to monitor and determine if actual antibiotic use and corresponding markers of antibiotic resistance go down [41]. The spread of antimicrobial resistance is very complex, and it is difficult to establish effective exposure assessments and dose-response models to try to more fully understand the risk that they pose to public health. While antibiotic stewardship is a positive step towards addressing this critical problem, the spread of resistance is also associated with person-to-person transmission, person-to-animal transmission (and vice versa), as well as the use of antimicrobial agents in consumer products. Development of a new QMRA framework that incorporates more detail on the physical, chemical, and biological mechanisms leading to resistance requires more research into many areas containing data gaps [38]. The more accurate and predictive our modeling can be, the better we can inform and prevent the development and spread of antibiotic resistance.

## Microbiology of the Built Environment: implications for building design

One theme that emerged throughout these presentations is that if we do not intentionally design for the indoor microbiome, then we become vulnerable to unintended consequences. This was most explicitly illustrated in Dr. Amy Pruden’s presentation on the premise plumbing microbiome where she highlighted studies demonstrating specific conditions that can promote growth of *L. pneumophila* [24, 25, 27–29]. We need to continue directed experiments such as those used in premise plumbing systems to determine specific factors that can be used to more broadly and intentionally control the indoor microbiome. In addition to water stagnation and water heater temperature, we should think towards other building design factors, such as ventilation, occupancy, and materials, to promote a healthy indoor microbiome. Steps in this process include both defining a healthy indoor microbiome and determining control measures to support relatively beneficial microbial populations. A healthy indoor microbiome has not yet been rigorously defined. Each indoor space harbors a unique microbial community that is temporally and spatially dynamic. Bacterial communities on surfaces and in air are dominated by microbes associated with human skin (and pets, if present) within most common microenvironments in homes, such as bedrooms and kitchens [42–44]. Indoor fungal communities are largely driven by outdoor fungal communities in buildings without moisture problems, and damp buildings often have distinct fungal communities that have been shown to have increased production of allergens, toxins, and pathogenicity [44, 45]. Building design and operation can greatly influence microbial communities. For example, the relative abundance of certain taxa are more prevalent during window ventilation periods than mechanical ventilation periods and vice versa [11].

## Panel discussion and future research directions

### Developing building standards

As MoBE research progresses, one beneficial outcome would be the establishment of new building standards to evaluate a building’s overall human health impact. These standards could then inform decisions by various entities involved in building design, construction, and management. The United States Green Building Council (USGBC) has developed Leadership in Energy and Environmental Design (LEED), which is a green building certification program that establishes a ranking system to evaluate different aspects of a building’s design, construction, operation, and maintenance [46]. Under the indoor environmental category, there are several different areas in which credits can be earned, including minimum indoor air quality, environmental tobacco smoke control, low-emitting materials, thermal comfort, interior lighting, and quality views [46].

There are not yet considerations in the LEED program given to microbial populations and the potential human health risk that they pose. Recent MoBE research indicates that there is cause for concern, for example the longer stagnation associated with water conservation can increase the potential for pathogen growth [47]. More research needs to be completed to ensure adoption of evidence-based standards related to the indoor microbiome. Research should focus on defining what constitutes a healthy indoor microbiome as well as effective measures to control microbial populations. Microbial communities can vary over time and depend largely on the humans occupying the space and how they make use of the different materials within the indoor environment [48]. Current standards and guidance exist now in other programs related more generically directed to moisture control [49], which is fundamentally known to be detrimental to building and occupant health.

The Hazard Analysis and Critical Control Point System (HACCP) is one approach that has been applied in the food industry to effectively evaluate potential hazards and their causes and effects [50]. Once hazards are identified, effective control mechanisms are put in place to ensure that future hazards are prevented from occurring. However, it is not clear whether the standard HACCP approach is appropriate for addressing MoBE concerns in buildings, as apparent in the current American Society of Heating, Refrigeration, and Air-Conditioning Engineers (ASHRAE) guidance for *Legionella* control, which has found a more general, building-specific approach to be optimal [51]. Applying a standard approach to the evaluation of the indoor environment within a building can allow for planning to prevent potential human health hazards, rather than waiting for problems to occur [50]. Given the amount of time humans spend indoors and the myriad of public health implications, a proactive approach is warranted in designing and maintaining buildings to promote healthy microbial exposures and/or reduce human exposures that potentially lead to disease. Defining what can be controlled within the indoor environment can help us engineer ways we can intervene to alleviate the problems. However, every building is different. Building assessment techniques that can be used to help establish healthy building standards need to be broad enough that they can be used in all buildings, and specific enough to help prevent potential exposures to health hazards.

### Policy considerations and liability concerns

The current practice in fields where microbiology and policy intersect, such as the food industry, utilizes indicator organisms to reflect the microbiological quality and safety from pathogens. These indicator organisms have qualities that meet specific criteria such as ease of measurement, enumeration in a short period of time, and a direct negative correlation with quality [52]. We do not currently have indicator organisms for many concerns in the built environment. However, we can still make informed decisions regarding indoor environmental quality. Water is a fundamental limiting factor for microbial growth and moisture content within indoor environments is complex. A non-invasive quantification of moisture content within buildings could become an innovative technology in locating microbial growth in buildings [53].

Once we are able to establish healthy building standards related to the indoor microbiome, we can then use them as a tool to improve the overall health of building occupants. This raises a possible concern for entities that are held legally responsible for building maintenance and cleanliness. Building owners and others would then be responsible for implementing procedures to be compliant to building standards, which could require extensive resources. There is also a conflict of interests for private cleaning companies between maintaining hygiene standards and cleanliness and receiving a profit [54]. Entities such as property owners that rent many units need incentives to improve indoor environmental quality. If not incentivized, there will likely be resistance to approving and implementing mandatory healthy building standards.

Policy detailing consequences for noncompliance as well as explicitly identifying liable parties in the occurrence of potential legal disputes would be required in building standards. Defining responsible parties can become difficult because buildings are complex and dynamic systems. There are factors that influence indoor environmental quality that are out of the building owner’s control. For instance, utilities such as water services that serve a building are reliant on outside entities before they reach a building. Depending on the situation, there may also be other standards to adhere to, such as the Clean Drinking Water Act. Establishing building regulations is a difficult situation to navigate, but the potential health risks associated with poor indoor environmental quality that can be averted with healthy building standards is motivation to continue to try.

### How clean is clean enough?

The hygiene hypothesis is an idea proposed by David P. Strachan in 1989 that was first received as a speculative explanation for the apparent rise in allergic diseases [55]. His findings suggested that children in larger households had fewer instances of hay fever, an allergic disease, because they are exposed to unhygienic microbial populations from older siblings [55]. Declining family size, improved amenities within the home and higher standards of personal hygiene have decreased childhood exposures to microbial populations. Less exposure makes it more difficult to build an immune response to common microbial populations, therefore increasing the expression of atopic disease [55]. When first introduced, his idea opposed the predominant view of microorganisms as harmful rather than potentially providing a protective effect. Since then, there have been studies that have expanded the idea to explain autoimmune diseases [56]. The hygiene hypothesis continues to be explored, although a rigorous definition has not yet emerged.

The hygiene hypothesis emphasizes that we may need to rethink our definition of “clean” to be an environment that is free of pathogenic organisms and contains beneficial species, as opposed to an environment that is sterile or entirely free of microorganisms. Thus, we need to define effective methods to measure microbial populations in indoor environments. Healthcare facilities deserve special consideration. Poor indoor environmental hygiene results in the transmission of microorganisms that cause hospital-acquired infections [54]. Exposures to detrimental microbial populations are a matter of life and death for the immunocompromised, neonates, elderly, and other vulnerable groups. Fortunately, there is already much interest in reducing nosocomial infections, which cost up to $45 billion in the USA each year [57]. Research in these buildings may be a concern for some owners’ due to fears associated with liability, but it is in the best interest of the owners and patients to improve these environments. As we become more aware of the microbial populations present in our buildings, people will increasingly demand that buildings be tested for pathogens and hold building owners responsible for controlling exposures to potential health risks.

The current cleaning and disinfecting strategies in hospitals vary largely depending on the surface. Methods can include low level disinfectants to simple soap-and-water cleaning; all of which need to be evaluated for necessary modifications to control the spread of pathogenic microorganisms [54]. Cleaning in hospitals serves two functions: the first is to keep the appearance of a clean environment that deters deterioration. The second function is to eliminate the microbial populations and remove growth material that would act as a reservoir for microorganisms [54]. Cleanliness can be assessed aesthetically, but that will not always ensure the environmental surface is clear of pathogenic organisms.

Often, standards to measure microbial cleanliness utilize certain indicator organisms. However, application of these methods may not always be thorough enough to assess cleanliness and are not always implemented in health care facilities [54]. Organisms that have acquired antibiotic resistance genes, such as methicillin-resistant *Staphylococcus aureus* (MRSA), are favored in hospital environments due to constant cleaning. Ill patient’s bacterial flora can become quickly dominated by these resistant organisms and can contaminate the environment near the patient [54, 58, 59]. The ability to verify the cleanliness of all environmental surfaces in hospitals is a major factor in eliminating the spread of these persistent organisms. Establishing cleanliness standards is a complicated task, but continued efforts are warranted to reduce hospital acquired infections and find ways to establish cooperation with researchers, hospital administration, and cleaning personnel to produce the healthiest indoor environment possible.

## Conclusions

This symposium highlighted recent research related to MoBE, including indoor exposures and childhood asthma, the influence of the hospital microbiome on neonatal development, the importance of the microbiome in premise plumbing systems, antibiotic resistance, and risk modeling. This research propels us ever closer to the development of microbiome-informed building standards to influence building design and promote occupant health. Future research needs broadly include defining a healthy indoor microbiome and exploring control measures. We need to be able to make building design choices to promote health or we are vulnerable to unintended consequences.

## Abbreviations

ARG: Antibiotic resistance genes
ASHRAE: American Society of Heating, Refrigeration, and Air-Conditioning Engineers
CAMRA: Center for Advancing Microbial Risk Assessment
HACCP: Hazard Analysis and Critical Control Point System
ICU: Intensive Care Unit
LEED: Leadership in Energy and Environmental Design
MoBE: Microbiology of the Built Environment
MRSA: Methicillin-resistant *Staphylococcus aureus*
NCSE: National Council for Science and the Environment
NICU: Neonatal Intensive Care Unit
NTM: Nontuberculous mycobacteria
QMRA: Quantitative microbial risk assessment
USGBC: United States Green Building Council
VBNC: Viable but non-culturable
